# What Is Evidence‐Based Pharmacy?

**DOI:** 10.1111/jep.70114

**Published:** 2025-05-19

**Authors:** Jennifer Maria Alexa, Katja Suter‐Zimmermann, Thilo Bertsche, Samuel S. Allemann

**Affiliations:** ^1^ Department of Pharmaceutical Sciences, Pharmaceutical Care Research Group University of Basel Basel Switzerland; ^2^ Department of Clinical Research, University Hospital Basel University of Basel Basel Switzerland; ^3^ Department of Clinical Pharmacy, Institute of Pharmacy, Faculty of Medicine Leipzig University Leipzig Germany

**Keywords:** definition, evidence‐based medicine, evidence‐based pharmacy, evidence‐based pharmacy practice, evidence‐based practice

**Abbreviations**
EBHC: evidence‐based healthcare
EBM: evidence‐based medicine
EBP: evidence‐based practice
EBPharm: evidence‐based pharmacy

*Evidence‐based pharmacy (EBPharm)* appears to be a vague term. This term has been used interchangeably and with overlapping meanings [1, 2, 3, 4, 5, 6, 7, 8, 9, 10, 11, 12, 13]. Furthermore, it remains underrepresented in the literature and seems to be mostly unknown [14, 15]. EBPharm has a great potential to contribute to an individualized, safe and effective pharmaceutical care and consequently to a reduced burden on healthcare systems. However, a successful implementation of EBPharm into practice requires a clear understanding of what it embodies. To date, a widely accepted definition of EBPharm is lacking. The purpose of this commentary is to propose a definition for EBPharm in alignment with existing terminology.

## 1 The Roots of Evidence‐Based Pharmacy and a Varying Terminology

EBPharm emerged in recent years based on *evidence‐based medicine (EBM)*. The most commonly used definition of EBM was coined in 1996 by D.L. Sackett and his colleagues [16]. They defined EBM as the ‘conscientious, explicit, and judicious use of current best evidence in making decisions about the care of individual patients.’ Within the same editorial the authors also stated that ‘integrating individual clinical expertise’ and the ‘patient's choice’ [16] are of great importance in EBM. Furthermore, according to Sackett et al. ‘good doctors use both individual clinical expertise and the best available external evidence,’ because ‘neither alone is enough [16].’

Despite the frequent use of this definition, some confusion and disagreement still seem to exist.

Many authors cited only the first part of the definition when referring to EBM. Therefore, EBM was mistakenly accused of neglecting health professionals' practical experiences and of being too focused on clinical data [17, 18]. However, it is well‐established that EBM integrates the following three factors equally: 1) external evidence as well as 2) a patient's preferences and 3) a healthcare professional's practical experience. This constitutes the core of EBM and other evidence‐based health disciplines. One could argue that the most commonly used sentence of the definition indirectly implies the consideration of all three factors. Misunderstandings may, nonetheless, be due to the fact, that Sackett et al. did not clearly mention all three key factors of EBM within their most prominent line of the editorial and subheading.

## 2 Overlap and Distinction Between Commonly Used Terms

Another cause for uncertainty and confusion is the ambiguous use of the term *evidence‐based practice (EBP)*. Some authors utilize EBP and EBM synonymously. Sackett et al. stated that ‘the practice of evidence‐based medicine’ requires ‘integrating individual clinical expertise with the best available external clinical evidence from systematic research’ [16].

Based on the Sicily statement on evidence‐based practice [19], however, EBP refers to the process of considering the three key factors in practice. EBP comprises 5 main steps, which are also often labelled as the 5‐step‐model [20]. The five main steps include: 1) The formulation of a precise clinical question, 2) a systematic search for the best available external evidence, 3) critical appraisal of the identified external evidence concerning internal and external validity, 4) transfer of results into practice in alignment with the patient's preferences and the healthcare professional's practical experience, and lastly 5) a performance evaluation concerning the effectiveness and safety of the intervention, if the patient revisits [19, 20]. These steps apply to all health disciplines. EBP is, therefore, independent of a health discipline. In contrast to EBP, the term *evidence‐based pharmacy practice* as used by C. Chant and H.Z. Toklu [8, 11] relates to EBP in the context of pharmacy.

The frequently cited definition of EBM by Sackett et al. originated in the medical context. As a result, different evidence‐based health disciplines, such as *evidence‐based nursing* or *evidence‐based physiotherapy*, were defined and established in recent years [21, 22]. This is due to the fact that each health profession's role in healthcare is substantially different based on the legal framework and scope of practice.

Despite profession‐related differences, evidence‐based health disciplines also share common grounds and often a multiprofessional collaboration. All evidence‐based health disciplines are united through the umbrella‐term *evidence‐based healthcare (EBHC)* [23, 24].

EBHC can be used to describe the concept of healthcare, that involves the consideration of the healthcare workforces' clinical expertise, the preferences and values of a patient (group) as well the best available external evidence when striving for best patient‐relevant outcomes. The term EBHC is, however, again used inconsistently and with overlapping meanings.

In general, EBHC aims to foster multiprofessional collaboration across all evidence‐based health disciplines. In this context, however, the need for a novel term evolved and manifested itself among evidence‐based healthcare‐oriented associations such as the “Deutsches Netzwerk Evidenzbasierte Medizin e.V.” [25] The term and abbreviation EBX is used to refer to any evidence‐based health discipline within all established evidence‐based health disciplines. The X stands for any unspecified health discipline. Box 1.

Box 1 **The definition of evidence‐based pharmacy.**

1 We suggest to define *evidence‐based pharmacy (EBPharm)* as a three‐factor‐based individualized concept that aims to achieve best patient‐relevant outcomes when counseling and making clinical decisions in the context of pharmacy. Evidence‐based pharmacy requires pharmacy staff to consider the following three factors:

1. the patient's preferences, values and circumstances;
2. their own practical experience (internal evidence) as well as;
3. the best available external evidence.

**Figure 1:**
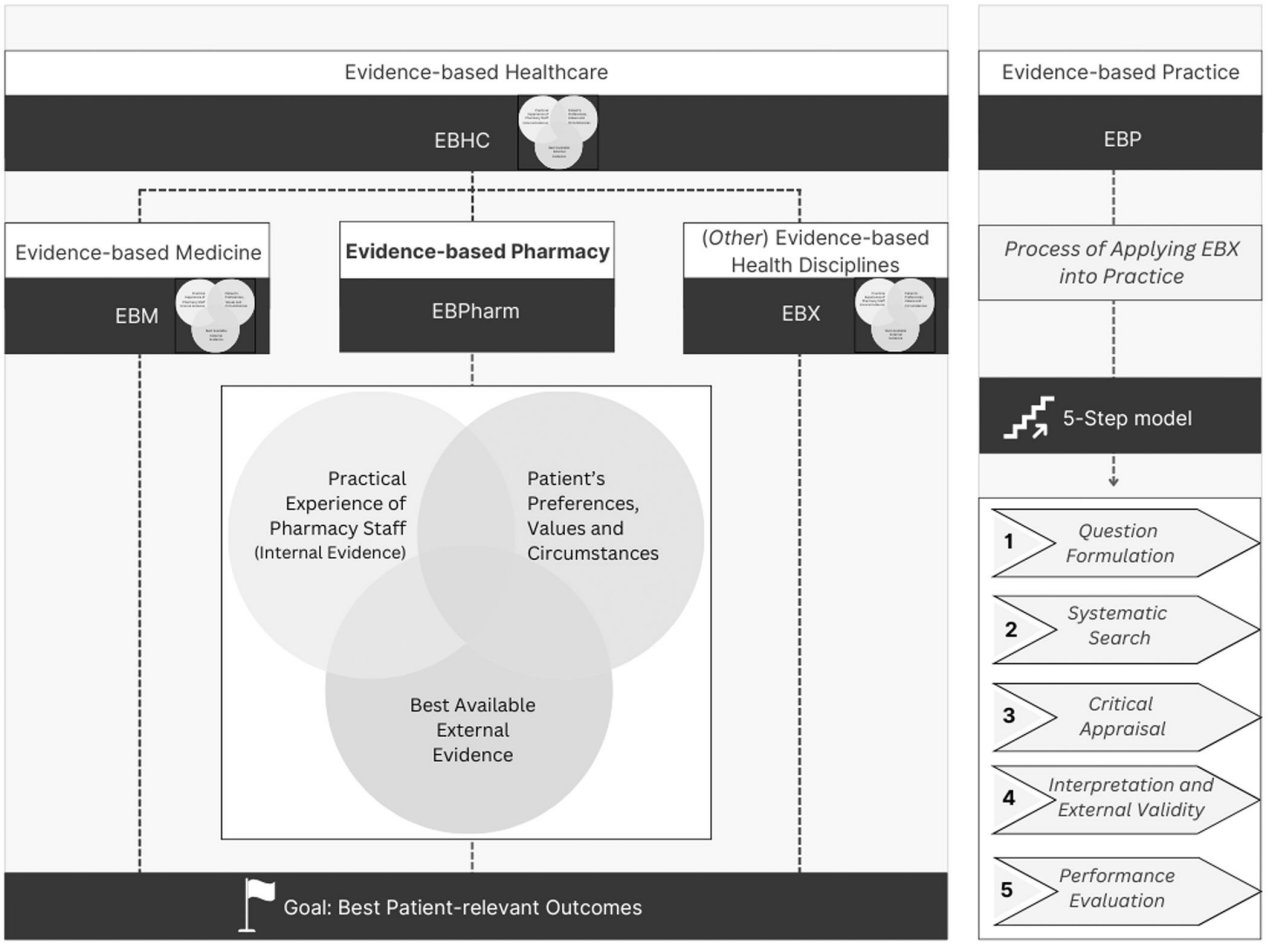
Graphical display of evidence‐based pharmacy and associated terms.

Figure 1 illustrates what evidence‐based pharmacy embodies as well as frequently used terms in relation to evidence‐based pharmacy.

The *best available external evidence* in this case refers to relevant data from clinical research that has been identified through a systematic search of available resources and is ideally of high internal validity as well as prone to a low risk of bias [26, 27]. *Internal evidence*, in contrast, describes each pharmacy staff's practical work experience, which accumulates over time and is shaped by feedback‐based interactions with patients for instance, own experiences with pharmaceutical interventions, and versatile expertise about pharmacology, medicine management, drug formulation or preparation for example. The best patient relevant outcome in this case refers to an outcome that is in alignment with the patient's preferences, values and circumstances. This requires an active involvement of the patient in the decision‐making process. EBPharm, therefore, also promotes *shared decision‐making* (SDM) between the pharmacist and the patient.

Finally, it is important to highlight that EBPharm is very dynamic and patient‐centered and does not at all mean to simply follow “cookbook” recommendations.

## 3 Implementing Evidence‐Based Pharmacy—The Next Steps

Continuous efforts are necessary to raise the awareness about EBPharm and EBHC. We recommend to use a consistent terminology related to EBPharm, EBHC, and EBP. A consistent terminology will ease the dissemination of pre‐existing knowledge and application of EBPharm into practice. This can ultimately contribute to achieving benefits on the patient‐level as well as for healthcare systems and research.

Moreover, this helps to avoid confusion and a loss of content. Existing research can, for instance, be adequately attributed to EBPharm and EBHC. Therefore, we also suggest to introduce EBPharm as a keyword and MESH‐term to help allocate pre‐existing research and to facilitate its accessibility. Previously published studies tended to focus on examining pharmaceutical staff's attitude towards evidence‐based medicine, barriers to evidence‐based medicine and evidence‐based practice, as well as the evaluation of evidence‐based medicine‐related educational interventions in the context of pharmacy [4, 28, 29, 30, 31].

By proposing a definition of EBPharm, we intend to contribute to a common understanding and to promote the consistent use of related terms. We ultimately hope to promote progress in the implementation of EBPharm into practice and pharmacy‐related research.

## Conflicts of Interest

The authors declare no conflicts of interest.

5

## Data Availability

The authors have nothing to report.
